# Radiation of meningioma dural tail may not improve tumor control rates

**DOI:** 10.3389/fsurg.2022.908745

**Published:** 2022-07-04

**Authors:** Keenan Piper, Siyuan Yu, Mohammad Taghvaei, Christian Fernandez, Nikolaos Mouchtouris, Rupert D. Smit, Clifford Yudkoff, Sarah Collopy, Maikerly Reyes, Pascal Lavergne, Michael Karsy, Giyarpuram N. Prashant, Wenyin Shi, James Evans

**Affiliations:** ^1^ Sidney Kimmel Medical College, Thomas Jefferson University Hospital, Philadelphia, PA, United States; ^2^ Department of Neurological Surgery, Thomas Jefferson University Hospital, Philadelphia, PA, United States; ^3^ Department of Radiation Oncology, Thomas Jefferson University Hospital, Philadelphia, PA, United States

**Keywords:** meningioma, dural tail, dural tail sign, radiotherapy, radiosurgery, SRS, FSRT, stereotacic radiation

## Abstract

**Introduction:**

Dural tails are thickened contrast-enhancing portions of dura associated with some meningiomas. Prior studies have demonstrated the presence of tumor cells within the dural tail, however their inclusion in radiation treatment fields remains controversial. We evaluated the role of including the dural tail when treating a meningioma with stereotactic radiation and the impact on tumor recurrence.

**Methods:**

This is a retrospective, single-institution, cohort study of patients with intracranial World Health Organization (WHO) grade 1 meningioma and identified dural tail who were treated with stereotactic radiosurgery (SRS) or fractionated stereotactic radiotherapy (FSRT) from January 2012 to December 2018. SRS and FSRT subgroups were categorized based on coverage or non-coverage of the dural tail by the radiation fields, as determined independently by a radiation oncologist and a neurosurgeon. Demographics, tumor characteristics, radiation plans, and outcomes were evaluated. High grade tumors were analyzed separately.

**Results:**

A total of 187 WHO grade 1 tumors from 177 patients were included in the study (median age: 62 years, median follow-up: 40 months, 78.1% female) with 104 receiving SRS and 83 receiving FSRT. The dural tail was covered in 141 (75.4%) of treatment plans. There was no difference in recurrence rates (RR) or time to recurrence (TTR) between non-coverage or coverage of dural tails (RR: 2.2% vs 3.5%, *P* = 1.0; TTR: 34 vs 36 months, *P* = 1.00). There was no difference in the rate of radiation side effects between dural tail coverage or non-coverage groups. These associations remained stable when SRS and FSRT subgroups were considered separately, as well as in a high grade cohort of 16 tumors.

**Conclusion:**

Inclusion of the dural tail in the SRS or FSRT volumes for meningioma treatment does not seem to reduce recurrence rate. Improved understanding of dural tail pathophysiology, tumor grade, tumor spread, and radiation response is needed to better predict the response of meningiomas to radiotherapy.

## Introduction

Meningiomas are the most common primary intracranial tumor, comprising about 25% of cranial lesions diagnosed in the United States (1). Despite surgical resection, stereotactic radiation, or a combination of the two, a significant subset of meningiomas recur. Published rates of recurrence range from 7%–25% for WHO grade I, 29%–59% for grade II, and 60%–94% for grade III tumors (2).

A dural tail sign (DTS) is a common finding seen with meningiomas that describes the thickening of the dura adjacent to a tumor on contrast enhanced T1 weighted MRI (3). The finding was first described in 1989 by Wilms et al. (4) and radiographic criteria for the phenomenon were subsequently developed by Goldsher et al. *i*n 1990 (5). Though not pathognomonic for meningiomas, prior studies have indicated that DTS has a diagnostic sensitivity of 58.6% and a specificity of 94.02% (6). Importantly, the rates of observed DTS vary by tumor location, more commonly occurring in falcine, tentorial, and convexity meningioma but less frequently in posterior fossa tumors (3).

Surgical resection and radiation treatment, namely stereotactic radiosurgery (SRS) and fractionated stereotactic radiotherapy (FSRT), are the most common meningioma treatments. Radiation provides an effective primary or adjuvant option for non-resectable, aggressive, or recurrent meningiomas as well as for residual tumor in the setting of subtotal resection (7). It is also often used in the management of small asymptomatic tumors that display growth on interval imaging. In a meta-analysis, Pinzi et al. estimated that 5-year disease control rates with SRS and hypo-fractionated FSRT range from 87 to 100% (8).

The clinical relevance of the dural tail and its inclusion as a target for radiotherapy remains contentious (9). In one large study, 88.3% of 179 dural tails resected from convexity meningiomas contained tumor cells. Notably, 95% of the cells existed within 2.5 cm of the tumor base (10). In an earlier series, 20 of 31 dural tails demonstrated tumor invasion (11). The Radiation Therapy Oncology Group (RTOG 0539) and European Organization for Research and Treatment of Cancer (EORTC 26021) studies suggest that nodular dural tails are more likely to contain aggressive disease as compared to smooth dural tails (12, 13). Although different tail geometries were equally likely to contain tumor cells, nodular tails were more common in higher-grade tumors (10). The presence of tumor cells in a large proportion of the dural tails would warrant its inclusion in the radiation treatment plan. However, including the tail can significantly increase the treatment volume and therefore the risk of complications (14). As a result, varying treatment recommendations exist with some providers opting for a larger field with dural tail inclusion while others opt for a smaller field to minimize the risk of complications.

Due to the lack of consensus guidelines, there is a pressing need to determine the benefits and risks of inclusion of the dural tail in the radiation treatment plan for meningiomas. In this study, we sought to analyze the outcomes related to inclusion and exclusion of the dural tail in SRS and FSRT isodose prescriptions in a single-institution retrospective cohort study.

## Methods

### Patient Selection, Variables, and Outcomes

The study protocol was reviewed and approved by the hospital Institutional Board Review and the need for informed consent was waived. Patient demographics, comorbidities, tumor location and characteristics, modality of radiation, history of tumor resection and radiation, rate of gross total resection (GTR), tumor recurrence, site of recurrence, radiation morbidities, and treatments were collected from the electronic health record. Radiation details were obtained from Mosaiq (Elekta, Stockholm, Sweden, v2.64), including isodose prescription, treatment volume, mean, and maximum dose volume. Indications for adjuvant therapy for World Health Organization (WHO) grade I and II tumors included subtotal resection or recurrence following initial surgery or radiation. If GTR was achieved for grade II meningioma, adjuvant treatment was case dependent.  All patients with grade III meningiomas received adjuvant radiotherapy.

Tumor recurrence was defined as a new area of enhancement on follow-up T1 contrast imaging determined by the radiology reports. The date of recurrence was determined by the first post-treatment MRI with demonstration of new tumor enhancement. To account for pseudoprogression and post-radiation changes, tumor growth had to be noted in two consecutive MRI scans. If there were equivocal radiographic findings, the results were adjudicated by a blinded 3rd neurosurgeon reviewer. Sites of recurrence were assessed based on comparison of immediate pre-radiation imaging and follow-up imaging. Symptomatic edema was defined by increasing T2/FLAIR hyperintensity on imaging with corresponding neurological symptoms mitigated by steroid treatment. Temporary neurological deficits were defined as neurological signs and symptoms lasting less than 6 months after radiation and permanent neurological deficits defined as signs and symptoms lasting more than 6 months. Radiation necrosis was defined by imaging, clinical symptoms, and histological findings corresponding with necrosis.

The Goldsher criteria were used on pre-operative magnetic resonance imaging (MRI) to determine presence or absence of dural tail (5). These include (1) the presence of two consecutive sections through the tumor at the same site in more than one imaging plane; (2) the greatest thickness adjacent to the tumor and tapering away; and (3) more intense enhancement than the tumor itself. Determination of dural tail inclusion or exclusion in treatment plans were made independently by a neurosurgeon and a radiation oncologist. Discrepancies were settled by a blinded 3rd physician.

### Inclusion/Exclusion Criteria

**Figure 1:**
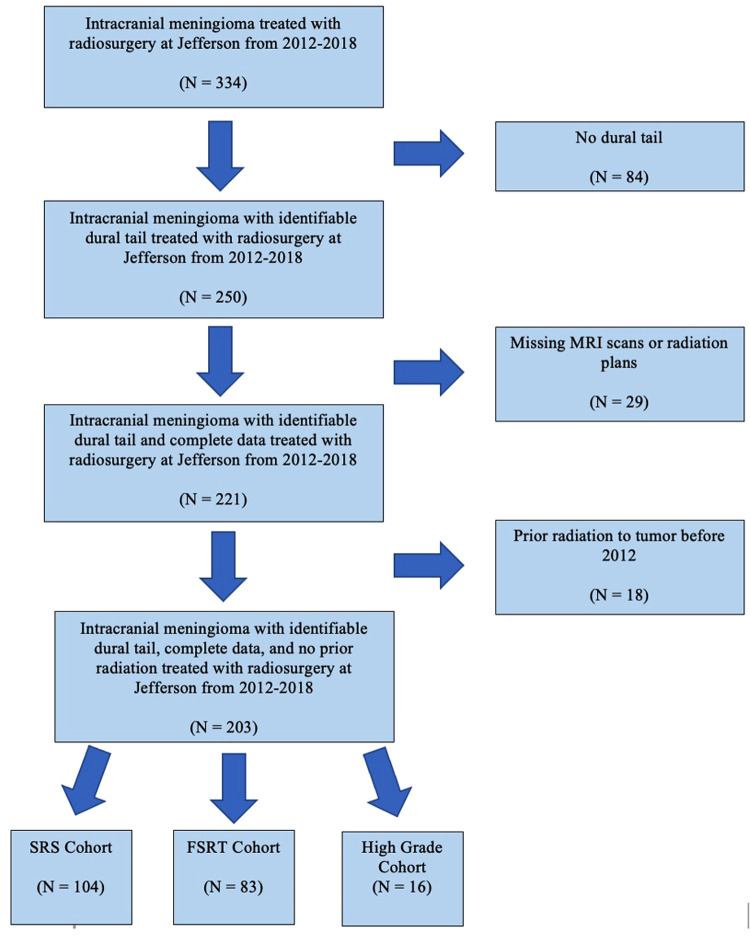
Flowchart of the inclusion criteria for this study.

Patients with an imaging diagnosis of meningioma who underwent SRS or FSRT by linear accelerator (LINAC) or gamma knife between January 2012 to December 2018 were screened (Figure 1). Only tumors with dural tail on the pre-operative MRI, as well as patients with adequate clinical, imaging and radiation planning therapy were included. Seventeen patients had received radiation prior to 2012 and were excluded from the study due to inability to access previous radiation records.

### Primary Outcome

The primary outcome of the study was to determine the rates of meningioma recurrence when comparing radiation plans that covered the dural tail to those without dural tail coverage in SRS and FSRT cohorts. Secondary outcomes were the rates of radiation-induced morbidities between the dural tail coverage and non-coverage groups in either cohort.

### Radiation Delivery

Patients were treated using the stereotactic linear accelerator (Varian 60°C before 2013; Varian Truebeam Stx after 2013) and Gammaknife (Elekta Gammaknife 4C unit before 2017; Elekta Gammaknife  iCON after 2018). Treatment planning was performed on the BrainLab iPLAN for LINAC and Elekta Leksell Gammaplan for gamma knife surgery.

Gross tumor volume (GTV) was defined as the contrast enhancing tumor on T1-weighted MR images for intact meningioma and the surgical tumor bed plus residual tumor for postoperative meningioma. Clinical target volume (CTV) was considered as GTV for WHO grade I meningioma. For WHO grade II or III and post-operative meningiomas, CTV was GTV plus a margin of 1–2 cm with adjustment of anatomical boundaries. The planning target volume (PTV) was define as CTV with a margin of 0–2 mm.  A median dose of 15 Gy single fraction for SRS, 25 Gy in 5 fractions for hypofractionated, and 54 Gy in 30 fractions (1.8 or 2 Gy/fraction) for FSRT was utilized. Hypofractionated radiotherapy and SRS were considered together.

### Follow-up

Follow-up visits included neurological evaluation and neuroimaging generally performed every 3–6 months for the first year and then annually/biannually based on tumor grade and surgeon preference.  Tumor response was assessed according to follow-up serial MRIs by a neurosurgeon and a neuroradiologist. Tumor sizes were compared with pre-treatment measurements to evaluate for tumor control or growth. Local tumor control was defined as the absence of radiological tumor recurrence. Date of recurrence was determined by the date of the first MRI which demonstrated tumor recurrence. Tumor recurrences were classified based on tumor growth origin either from the bulk tumor or dural tail segment. Radiation treatment plans accounting for dural tail coverage and recurrence were reviewed by a radiation oncologist and a neurosurgeon. Cases where coverage was unclear were reviewed by a blinded 3rd neurosurgeon reviewer. Rate of recurrence, time to recurrence, and radiation-induced morbidities were compared between patients with or without dural tail targeting. Follow-up was defined by the date of the last brain MRI. Recurrent tumors were assessed for area of recurrence and were classified as (1) local (recurrence within a treatment field), (2) marginal (recurrence within 1 cm outside of the treatment field), and (3) distant (recurrence 1 cm outside a treatment field).

### Statistical Analysis 

All continuous parametric data are reported as means with standard deviation and all non-parametric data are reported as medians with 25th and 75th quartiles. Categorical variables were compared using chi-square analysis or Fisher's exact test. Student t-test was used to analyze continuous variables and Mann-Whitney U test was used to analyze nonparametric data. Statistical significance was considered as *P* < 0.05. Kaplan Meier curves were generated to examine recurrence-free survival from tumor recurrence. Statistical analysis was carried out with IBM SPSS (Version 27.0. Armonk, NY: IBM Corp.). 

## Results

A total of 334 screened patients with intracranial meningioma underwent radiotherapy (Figure 1). Among these, 163 were eliminated, including 29 patients with missing pre-radiation MRI or radiation plans, and 84 patients lacking a dural tail on pre-radiation MRI. Eighteen tumors were eliminated because they had received prior radiation to that tumor before our study start date. A total of 187 WHO grade 1 intracranial meningiomas from 177 patients were included in our study. Of the 187 tumors, 119 meningiomas received radiosurgery as a primary treatment, in where the diagnosis of meningioma was made based on MRI criteria (3). The remaining 68 radiated meningiomas had undergone previous surgery with histologically confirmed meningioma. Grade 1 meningioma were further broken down into SRS and FSRT cohorts. Sixteen high grade (WHO grade 2 and 3) meningioma were analyzed separately.

### Grade 1 Meningioma Cohort

The SRS and FSRT cohorts were considered together with a total of 187 WHO grade 1 tumors. The median age of the entire group was 62 years (IQR: 52–70), with 146 (78.1%) females (Table 1). 68 (36.4%) patients underwent resection and had a histological diagnosis of meningioma prior to receiving radiation, whereas 119 (62.6%) had radiographically diagnosed meningioma only. Among patients receiving radiation following tumor resection, 19 (27.9%) of the cases had a GTR. In each of these cases, the radiation was given for treatment of recurrent tumor. Skull base tumors comprised 101 (54.0%) of the tumors. When stratified by location, the most frequent tumor locations were: convexity (*N* = 31, 17.6%), cavernous sinus (*N* = 22, 11.8%), parasagittal (*N* = 20, 10.7%), petroclival (*N* = 20, 9.0%) and medial sphenoid wing (*N* = 14, 7.5%). Of the 52 tumors with known Ki-67, the median index was 3.5 (IQR: 2.18–7.15). Median tumor volume was 5.2 cm^3^ (2.5–14.3 cm^3^) (Table 1).

**Table 1:** Demographics of 177 patients with 187 radiation treated grade 1 meningiomas.

	Total (*N* = 187)	Dural tail not covered (*N* = 46)	Dural tail covered (*N* = 141)	*P*-value
Age (interquartile range)	62 (52–70)	66 (57–72)	59 (51–69)	**0** **.** **009**
Female	146 (78.1%)	39 (84.8%)	107 (75.9%)	0.14
Race and Hispanic ethnicity				0.62
White	155 (82.9%)	36 (78.3%)	119 (84.4%)	
Black	23 (12.3%)	8 (17.4%)	15 (10.6%)	
Asian	6 (3.2%)	1 (2.2%)	5 (3.5%)	
Hispanic	3(1.4%)	1 (2.2%)	2 (1.4%)	
Comorbidities				
None	27 (14.4%)	3 (6.5%)	24 (17.0%)	0.09
Diabetes	59 (31.6%)	17 (37.0%)	42 (29.8%)	0.37
Hypertension	37 (19.8%)	5 (10.9%)	32 (22.7%)	0.09
Cardiac	27 (14.4%)	6 (13%)	21 (14.9%)	1.00
Radiosurgery	104 (55.6%)	35 (76.1%)	69 (48.9%)	**0**.**002**
Radiotherapy	83 (44.4%)	11 (23.9%)	72 (51.1%)	
Resection prior to radiation	68 (36.4%)	13 (28.3%)	55 (39.0%)	0.22
Gross total resection, *N* = 68	19 (27.9%)	6 (46.3%)	13 (23.6%)	0.10
Location				
Falx	18 (9.6%)	3 (6.5%)	15 (10.6%)	0.57
Convexity	33 (17.6%)	22 (47.8%)	11 (7.8%)	**0**.**0001**
Parasagittal	20 (10.7%)	6 (13.0%)	14 (9.9%)	0.59
Cavernous sinus	22 (11.8%)	1 (2.2%)	21 (14.9%)	**0**.**018**
Petroclival	20 (9.0%)	1 (2.2%)	17 (12.1%)	0.079
Foramen magnum	4 (1.8%)	2 (4.3%)	2 (1.4%)	0.25
CPA	11 (5.9%)	1(2.2%)	10 (7.1%)	0.30
Sphenoid wing				
Lateral	4 (2.1%)	2 (4.3%)	2 (1.4%)	0.45
Medial	14 (7.5%)	4 (8.7%)	10 (7.1%)	
Skull base tumors	101 (54.0%)	11 (23.9%)	90 (63.8%)	**0**.**0001**
Ki 67, *N* = 52 (median, Quartile)	3.5 (2.18–7.15)	4.0 (1.5–8.4)	3.4 (2.3–6.8)	0.82
Volume, CM^3^ (median, Quartile)	5.2 (2.5–14.3)	3.3 (0.93–7.1)	11.7 (5.1–26.2)	**0**.**0001**

*The bold means results are statistically significant at a p < 0.05.*

The median follow-up for the entire cohort was 40 months (22–59) and local tumor control was achieved in 96.8% of the patients. Recurrence was detected in 6 (3.2%) of the grade 1 tumors and the median time to recurrence was 35 months (IQR: 25.8–41.0). The most common side effects from radiation were headaches in 31 (16.6%) patients followed by symptomatic edema in 15 (8.0%) patients. Radiation necrosis occurred in 6 (3.2%) patients and permanent neurological deficits were seen in 2 (1.1%) patients. Steroids were prescribed to manage post radiation symptoms in 30 (16.0%) patients and antiepileptics were used in 16 (8.6%) patients. Surgery was required in 5 (2.7%) cases for management of symptomatic edema/radiation necrosis. Zero patients required Bevacizumab for treatment of radiation necrosis (Table 2). 

**Table 2:** Primary outcomes for coverage vs. non-coverage of dural tail in grade I meningioma.

	Total (*N* = 187)	Dural tail not covered (*N* = 46)	Dural tail covered (*N* = 141)	*P*-value
Tumor recurrence	6 (3.2%)	1 (2.2%)	5 (3.5%)	1.00
Recurrence site				
Local	2	0	2	
Marginal	1	0	1	
Distant	3	1	2	
Median time to recurrence (months)	35 (25.8–41.0)	34	36 (25–44)	1.00
Radiation Morbidities				
Headache	31 (16.6%)	4 (8.7%)	27 (19.1%)	0.11
Seizure	10 (5.3%)	3 (6.5%)	7 (5.0%)	0.71
Asymptomatic edema	11 (5.9%)	2 (4.3%)	9 (6.4%)	1.00
Symptomatic edema	15 (8.0%)	7 (15.2%)	8 (5.7%)	0.06
Radiation necrosis	6 (3.2%)	1 (2.2%)	5 (3.5%)	1.00
Temporal neurological deficit	12 (6.4%)	4 (8.7%)	8 (5.7%)	0.49
Permanent neurological deficit	2 (1.1%)	2(4.3%)	0 (0%)	0.06
Treatment for Radiation Morbidities				
Steroids	30 (16.0%)	9 (19.6%)	21 (15%)	0.49
Antiepileptics	16 (8.6%)	7 (15.2%)	9 (6.4%)	0.075
Surgery	5 (2.7%)	3 (6.5%)	2 (1.4%)	0.098
Bevacizumab	0 (0%)	0 (0%)	0 (0%)	0
Median follow up	40 (22–59)	35 (18.8–61.5)	41 (23–57.5)	0.96

When stratified by radiation treatment plans, 46 (24.6%) tumors had dural tail non-covered plans and 141 (75.4%) tumors had dural tail covered plans (Table 1). Dural tail non-covered patients were significantly older than dural tail covered patients (median age 66 vs 59 years; *P* = 0.009). There were no significant difference in sex, race, or comorbidities between the two groups. Additionally, there were no differences in history of resections prior to radiation, rate of GTR, or median KI-67 index. 

The non-covered dural tail group received significantly higher rates of SRS (76.1% vs 48.9%; *P* = 0.002). Dural tail covered patients had higher rates of skull base tumors (63.8% vs 23.9%; *P* = 0.0001). When examined by specific tumor location, cavernous sinus tumors were more frequent in the dural tail covered group (14.9% vs 2.2%, *P* = 0.018), whereas the non-covered group had higher rates of convexity tumors (47.8% vs. 7.8%, *P* = 0.0001). There was a significant difference in tumor volume (median volume: 3.3 vs 11.7 cm^3^; *P* = 0.0001) between non-covered and covered dural tails. Non-dural tail covered patients were more often treated with SRS (76.1% vs 48.9%, *P* = 0.002) whereas dural tail covered patients were more commonly treated with FSRT (51.1% vs 23.9%, *P* = 0.002).

**Figure 2:**
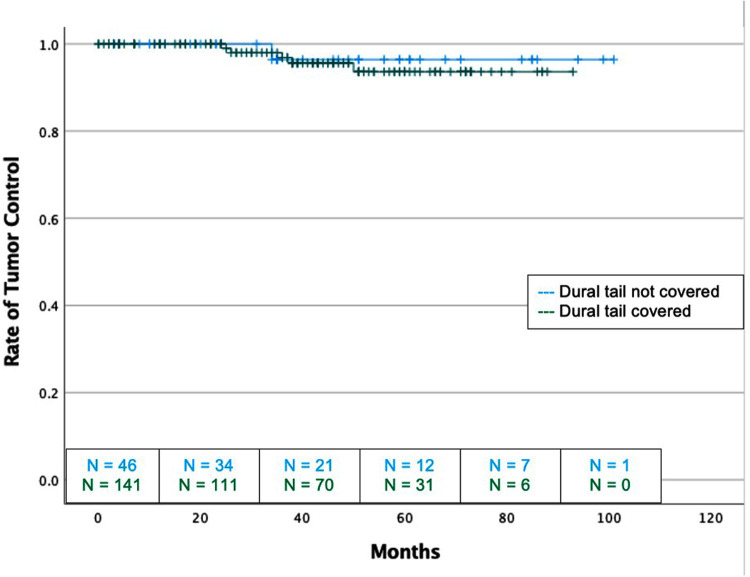
Kaplan-Meier graph demonstrating rate of tumor control or dural tail covered and not covered tumors with SRS and FSRT cohorts considered together. Blue: Dural tail not covered, Green: Dural tail covered. Dashes indicate censored patients due loss of follow-up or death. Number of patients remaining without recurrence or loss to follow-up is provided along the x-axis at 20-month intervals (Log Rank *P* = 0.723).

There was no difference in recurrence rates between dural tail non-covered versus dural tail covered groups (2.2% vs 3.5%; *P* = 1.00) or in time to recurrence (median: 34 vs 36 months, *P* = 1.00) (Table 2). There was no difference in radiation related morbidities including headaches, seizures, symptomatic edema, asymptomatic edema, radiation necrosis, temporary or permanent neurological deficits. There was no difference in treatment for radiation morbidities between the two groups. Additionally, no difference in tumor recurrence free survival was seen between two groups (Log Rank *P* = 0.631) (Figure 2). 

Of the 6 recurrent grade 1 tumors, 5 (83.3%) occurred in the dural tail covered group, and 1 (16.7%) occurred in the non-covered group. One of the 6 recurrences (16.7%) were marginal, 2 (33.4%) recurred locally, and 3 (50.0%) recurred in a distant area (Table 2). All 3 of the tumors which recurred locally or marginally were part of the dural tail covered group. There were no instances of a local recurrence in a tumor that did not have the dural tail covered and no tumors recurred from an untreated dural tail. Two recurrences were treated with further SRS, two were treated with FSRT, and two were treated surgically.

### Grade 1 SRS Subgroup

A total of 104 patients with WHO grade 1 meningioma received unfractionated or hypofractionated stereotactic radiosurgery (SRS). The median age of the included patients was 66 years (interquartile range (IQR): 54–73) with 78 (75%) females (Table 3). 33 (31.7%) patients underwent resection and had a histological diagnosis of meningioma prior to receiving radiation, whereas 71 (68.3%) had radiographically diagnosed meningioma only. Among patients receiving radiation following tumor resection, 16 (47.1%) cases had GTR. In each of these cases, the radiation was given for treatment of recurrent tumor. Skull base tumors comprised 30 (28.8%) of the tumors. When stratified by location, the most frequent tumor locations were convexity (*N* = 29, 27.9%), falx (*N* = 16, 15.4%), parasagittal (*N* = 16, 15.4%), and cerebellopontine angle (*N* = 6, 5.8%). Of the 24 tumors with known Ki-67, the median index was 4 (IQR: 3–9). The median isodose prescription for all tumors was 83% (IQR: 75%–90%) and median tumor volume was 3.3 cm^3^ (IQR: 1.5–6.9 cm^3^).

**Table 3:** Grade 1 meningioma SRS cohort demographics and outcomes.

	Total = 104	Stereotactic radiation (SRS) with Dural tail not covered (*N* = 35)	Stereotactic radiation (SRS) with Dural tail covered (*N *= 69)	*P*-value
Age, median (interquartile range)	66 (54–73)	69 (64–81.0)	61 (52–72)	0.30
Sex (female)	78 (75%)	29 (82.9%)	49 (71.0%)	0.24
Race and Hispanic ethnicity				0.156
White	94 (90.4%)	29 (82.9%)	65 (94.2%)	
Black	8 (7.7%)	8 (11.4%)	4 (5.8%)	
Asian	1 (1.0%)	1 (2.9%)	0 (0%)	
Hispanic	1 (1.0%)	1 (2.9%)	0 (0%)	
PMH				
None	17 (16.3%)	2 (5.7%)	15 (21.7%)	**0** **.** **049**
Diabetes	34 (32.7%)	13 (37.1%)	21 (30.4%)	0.51
HTN	21 (20.2%)	4 (11.4%)	17 (24.6%)	0.13
Cardiac	16 (15.4%)	3 (8.6%)	13 (18.8%)	0.25
Location				
Falx	16 (15.4%)	3 (8.6%)	13 (18.8%)	0.14
Convexity	29 (27.9%)	21 (60.0%)	8 (11.6%)	**0**.**001**
Parasagittal	16 (15.4%)	6 (17.1%)	10 (14.5%)	0.78
Cavernous sinus	0 (0%)	0 (0%)	0 (0%)	
Petroclival	4 (3.8%)	1 (2.9%)	3 (4.3%)	1.00
Foramen magnum	0 (0%)	0 (0%)	0 (0%)	
CPA	6 (5.8%)	0 (0%)	6 (8.7%)	0.095
Sphenoid wing				
Lateral	3 (2.9%)	0 (0%)	4 (5.8%)	0.35
Medial	4 (3.8%)	1 (2.9%)	2 (2.9%)	
Gross total resection	16 (47.1%)	6 (66.7%)	10 (40.0%)	0.25
Resection prior to radiation	33 (31.7%)	9 (25.7%)	24 (34.8%)	0.38
Skull-base tumors	30 (28.8)	1 (2.9%)	29 (42%)	**0**.**0001**
KI-67 (*N* = 24)	4 (3–9)	4 (0–7.4)	4 (3.0–9.7)	0.84
Volume radiation field (cm^3^)	3.3 (1.5–6.9)	3.2 (0.75–3.9)	7.4 (4.4–11.6)	0.07
Isodose prescription	83 (75–90)	83.0 (70–85)	85 (80–98)	0.62
Median follow-up (months)	37 (19–59)	59 (20–71)	30 (17–50)	0.54
Recurrence	2 (1.9%)	1 (2.9%)	1 (1.4%)	1.00
Time to recurrence	36 (34–38)	34	38	1.00
Recurrence site				
Local		0	0	
Marginal		0	1	
Distant		1	0	
Radiation Morbidities				
Headache	6 (5.8%)	1 (2.9%)	5 (7.2%)	0.66
Seizure	6 (5.8%)	3 (8.6%)	3 (4.3%)	0.40
Asymptomatic edema	4 (3.8%)	1 (2.9%)	3 (4.3%)	1.00
Symptomatic edema	12 (11.5%)	6 (17.1%)	6 (8.7%)	0.21
Radiation necrosis	6 (5.8%)	1 (2.9%)	5 (7.2%)	0.66
Temporal neurological deficit	6 (5.8%)	4 (11.4%)	2 (2.9%)	0.18
Permanent neurological deficit	2 (1.9%)	2 (5.7%)	0 (0%)	0.11
Treatment for Radiation Morbidities				
Steroids	17 (16.5%)	8 (22.9%)	9 (13.2%)	0.27
Antiepileptics	11 (10.7%)	7 (20.0%)	4 (5.9%)	**0**.**04**
Surgery	5 (4.9%)	3 (8.6%)	2 (2.9%)	0.33
Bevacizumab	0 (0%)	0 (0%)	0 (0%)	** **

*The bold means results are statistically significant at a p < 0.05.*

The median follow-up for the SRS cohort was 37 months (IQR: 19–59) and local tumor control was achieved in 98.1% of the patients. Recurrence was detected in 2 (1.9%) of the grade 1 tumors and the median time to recurrence was 36 months (range: 34–38). The most common side effects from radiation were symptomatic edema in 12 (11.5%) patients followed by headaches, seizure, and radiation necrosis in 6 (5.8%) patients each. Permanent neurological deficits were seen in 2 (1.9%) patients. Steroids were prescribed to manage post radiation symptoms in 17 (16.5%) patients and antiepileptics were used in 11 (10.7%) patients. Surgery was required in 5 (4.9%) cases for management of symptomatic edema/radiation necrosis. No patient required Bevacizumab for treatment of radiation necrosis (Table 3).

When stratified by radiation treatment plans, 69 (66.3%) tumors had radiographic dural tail covered and 35 (33.7%) tumors had a radiographic dural tail that was not covered (Table 3). Dural tail covered patients were more likely to have no significant past medical history (5.7% vs. 21.7%, *P* = 0.049). There were no significant difference in age, sex, race, or specific comorbidities between the two groups. Additionally, there were no differences in resections prior to radiation, rate of GTR, median KI-67 index, median follow-up, isodose prescription, or tumor size. Dural tail non-covered tumors were more commonly located on the convexity (60.0% vs. 11.6%, *P* = 0.001) and were less commonly skull-base (2.9% vs 42.0%, *P* = 0.0001).

**Figure 3:**
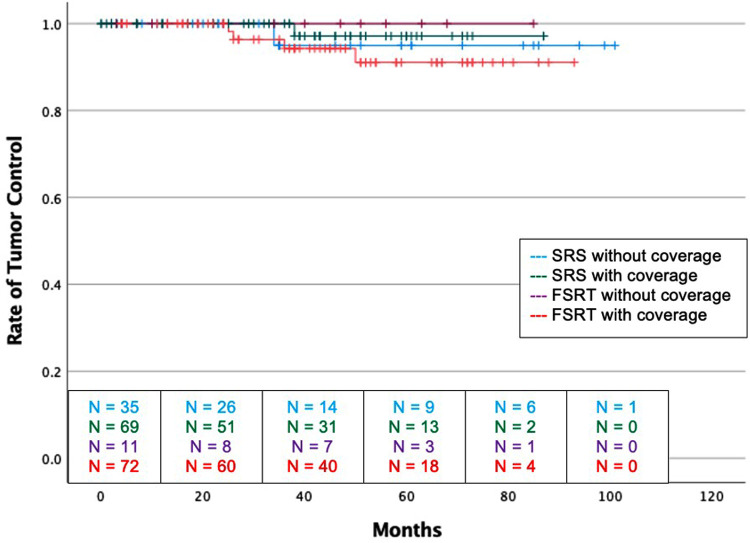
Kaplan-Meier graph demonstrating rate of tumor control amongst the four groups. Blue: SRS without dural tail coverage, Green: SRS with dural tail coverage, Purple: FSRT without dural tail coverage, Red: FSRT with dural tail coverage. Dashes indicate censored patients due loss of follow-up or death. Number of patients remaining without recurrence or loss to follow-up is provided along the x-axis at 20-month intervals (Log Rank *P* = 0.631).

There was no difference in recurrence rates between dural tail non-covered versus dural tail covered groups (1.9% vs 2.9%; *P* = 1.00) or time to recurrence (34 vs. 38 months, *P* = 1.00) (Table 3). Likewise, there was no difference in rates of post-radiation headache, seizure, edema, radiation necrosis, or neurological deficits between the two groups. The dural tail non-covered group more frequently required antiepileptics after radiation (20.0% vs 5.9%; *P* = 0.04). There was no difference in radiation related morbidities including headaches, seizures, asymptomatic edema, radiation necrosis, temporary or permanent neurological deficits. Additionally, no difference in tumor recurrence free survival was seen between the two groups (Log-Rank *P* = 0.631) (Figure 3). 

Of the 2 recurrent tumors, 1 occurred in the dural tail covered group, and 1 occurred in the non-covered group. The dural tail covered recurrence occurred marginally while the dural tail non-covered recurrence occurred distantly (Table 3). No tumors recurred from an untreated dural tail.

### Grade 1 FSRT Subgroup

A total of 83 patients with WHO grade 1 meningioma received fractionated stereotactic radiotherapy (FSRT). The median age of the included patients was 56 years (IQR: 48–66) with 68 (81.9%) females (Table 4). 35 (42.2%) patients underwent resection and had a histological diagnosis of meningioma prior to receiving radiation, whereas 48 (57.8%) had radiographically diagnosed meningioma only. Among patients receiving radiation following tumor resection, 3 (8.8%) of the cases had a GTR. In each of these cases, the radiation was given for treatment of recurrent tumor. Skull base tumors comprised 71 (85.5%) of the tumors. When stratified by location, the most frequent tumor locations were: cavernous sinus (*N* = 22, 26.5%), petroclival (*N* = 14, 16.9%), and medial sphenoid wing (*N* = 10, 12%). Of the 28 tumors with known Ki-67, the median index was 3.2 (IQR: 1.5–5.8). The median isodose prescription for all tumors was 98% (IQR: 93–98%) and median tumor volume was 13.8 cm^3^ (IQR: 5.0–25.0 cm^3^).

**Table 4:** Grade 1 meningioma FSRT cohort demographics and outcomes.

	Total = 83	Stereotactic radiotherapy (FSRT) with Dural tail not covered (*N* = 11)	Stereotactic radiotherapy (FSRT) with Dural tail covered (*N* = 72)	*P*-value
Age, median (interquartile range)	56 (48–66)	63 (59.5–77)	49 (42.0–58.0)	0.15
Sex (female)	68 (81.9%)	10 (90.9%)	58 (80.6%)	0.68
PMH				
None	10 (12.0%)	1 (9.1%)	9 (12.5%)	1.00
DM	25 (30.1%)	4 (36.9%)	21 (29.2%)	0.73
HTN	16 (19.3%)	1 (9.1%)	15 (20.8%)	0.68
Cardiac	11 (13.3%)	3 (27.3%)	8 (11.1%)	0.16
Race and Hispanic ethnicity				
White	62 (74.7%)	7 (63.6%)	55(76.4%)	0.324
Black	15 (18.1%)	4 (36.4%)	11 (15.3%)	
Asian	5 (6.0%)	0 (0%)	5 (6.9%)	
Hispanic	1 (1.2%)	0 (0%)	1 (1.4%)	
Location				
Falx	2 (2.4%)	0 (0%)	2 (2.8%)	1.00
Convexity	4 (4.8%)	1 (9.1%)	3 (4.2%)	0.44
Parasagittal	4 (4.8%)	0 (0%)	4 (5.6%)	1.00
Cavernous sinus	22 (26.5%)	1 (9.1%)	21 (29.2%)	0.27
Petroclival	14 (16.9%)	0 (0%)	14 (19.4%)	0.20
Foramen magnum	4 (4.8%)	2 (18.2%)	2 (2.8%)	0.083
CPA	5 (6.0%)	1 (9.1%)	4 (5.6%)	0.52
Sphenoid wing				
Lateral	1 (1.2%)	1 (9.1%)	0(0%)	**0**.**001**
Medial	10 (12%)	4 (36.4%)	6 (8.3%)	** **
Gross total resection	3 (8.8%)	0 (0%)	3 (10.0%)	1.00
Resection prior to radiation	35 (42.2%)	4 (36.4%)	31 (43.1%)	0.75
Ki-67*(28)	3.2 (1.5–5.8)	3.1 (2.6–13.1)	3.3 (1.3–5.0)	0.57
Skull-base tumors	71 (85.5%)	10 (90.9%)	61 (84.7%)	1.00
Volume radiation field (cm^3^)	13.8 (5.0–25.0)	7.0 (5.1–44.5)	20.5 (9.9–31.7)	**0**.**03**
Isodose prescription	98 (93–98)	96 (90.0–98.0)	98 (96.0–98.0)	0.09
Median follow-up (months)	45 (25–63)	10 (6.5–22.0)	52 (38–67)	0.84
Recurrence	4 (4.8%)	0 (0%)	4 (5.6%)	1.00
Time to recurrence	33 (26–40)	N/A	33 (26–40)	
Recurrence site				
Local		0	2	
Marginal		0	0	
Distant		0	2	
Radiation Morbidities				
Headache	25 (30.1%)	3 (27.3%)	22 (30.6%)	1.00
Seizure	4 (4.8%)	0(0%)	4 (5.6%)	1.00
Asymptomatic edema	7 (8.4%)	1 (9.7%)	6 (8.3%)	1.00
Symptomatic edema	2 (2.8%)	1 (9.1%)	2 (2.8%)	0.35
Radiation necrosis	0 (0%)	0 (0%)	0 (0%)	1.00
Temporal neurological deficit	6 (7.2%)	0 (0%)	6 (8.3%)	1.00
Permanent neurological deficit	1 (1.4%)	0 (0%)	1 (1.4%)	1.00
Treatment for Radiation Morbidities				
Steroids	13 (15.7%)	1 (9.1%)	12 (16.7%)	1.00
Antiepileptics	5 (6.0%)	0 (0%)	5 (6.9%)	1.00
Surgery	0 (0%)	0 (0%)	0 (0)	
Bevacizumab	0 (0%)	0 (0)	0 (0)	

*The bold means results are statistically significant at a p < 0.05.*

The median follow-up for the FSRT cohort was 45 months (IQR: 25–63 months) and local tumor control was achieved in 95.2% of the patients. Recurrence was detected in 4 (4.8%) of the tumors and the median time to recurrence was 33 months (IQR: 26–40). The most common side effects from radiation were headaches in 25 (30.1%) patients followed by asymptomatic edema in 7 (8.4%) patients. Radiation necrosis occurred in zero patients and permanent neurological deficits were seen in 1 (1.4%) patient. Steroids were prescribed to manage post radiation symptoms in 13 (15.7%) patients and antiepileptics were used in 5 (6.9%) patients. Neither surgery nor Bevacizumab were required for management of post-radiation complications.

When stratified by radiation treatment plans, 72 (86.7%) tumors had radiographic dural tail covered and 11 (13.3%) tumors had a radiographic dural tail that was not covered (Table 4). There were no significant differences in age, sex, race, or comorbidities between the two groups. Additionally, there were no differences in resections prior to radiation, rate of GTR, or median KI-67 index. Tumors in the dural tail covered group were significantly larger than those in the non-covered group (median volume: 20.5 vs 7.0 cm^3^, *P* = 0.03)

There was no difference in recurrence rates between dural tail non-covered versus dural tail covered groups (0.0% vs 5.6%; *P* = 1.00) (Table 4). There was no difference in radiation related morbidities including headaches, seizures, symptomatic edema, asymptomatic edema, radiation necrosis, temporary or permanent neurological deficits. There was also no difference in treatment for radiation morbidities. Additionally, no difference in tumor recurrence free survival was seen between two groups (Log-Rank *P* = 0.631) (Figure 3).

All 4 of the recurrent tumors occurred in the dural tail covered group. 2 of the 4 recurrences (50.0%) were local and 2 (50%) recurred in a distant area (Table 4). No tumors recurred from an untreated dural tail.

### Comparison of SRS vs. FSRT

Between SRS and FSRT cohorts, no significant differences were seen in age, gender, rate of GTR, resection prior to radiation, prior cranial radiation, KI-67 indexes, volume of the radiation field, isodose prescription, max dose, mean dose, median follow-up duration, radiation morbidities, tumor recurrence, or recurrence free survival.

### Grade II and III Meningioma Cohort

**Figure 4:**
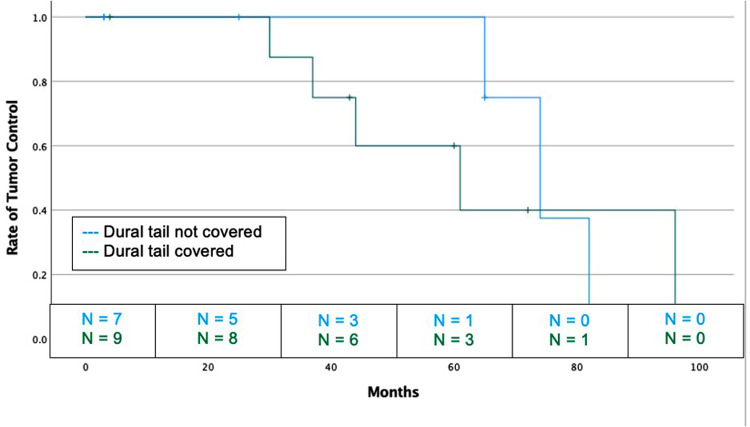
Kaplan-Meier graph demonstrating rate of tumor control or dural tail covered and not covered tumors for high grade (WHO grade 2 and 3) tumors. Blue: Dural tail not covered, Green: Dural tail covered. Dashes indicate censored patients due loss of follow-up or death. Number of patients remaining without recurrence or loss to follow-up is provided along the x-axis at 20-month intervals (Log Rank *P* = 0.670).

Sixteen grade 2 and 3 meningioma were analyzed as their own high-grade cohort (Table 5). The dural tail was covered in 9 tumors (56.3%) and not covered in 7 (43.6%). There was no difference between coverage and non-coverage in regards to age, sex, prior resection, rate of GTR, Ki-67, tumor location, tumor size, rates of SRS vs FSRT, or median follow-up. At a median of 52 months, there was no difference rate of recurrence between dural tail non-covered and covered groups (42.9% vs. 55.6%, *P* = 1.00) (Figure 4).

**Table 5:** Grade 2 and 3 cohort demographics and outcomes.

	Total = 16	Dural tail not covered, (*N* = 7)	Dural tail covered, (*N* = 9)	*P*-value
Age, median (interquartile range)	60 (53–65)	57(54–65)	62 (48–77)	0.68
Sex (female)	10 (62.5%)	6 (85.7%)	4 (44.4%)	0.16
WHO grade				
Grade II	15 (93.8%)	6 (85.7%)	9 (100%)	0.44
Grade III	1 (6.3%)	1 (14.3%)	0 (0%)	
Gross total resection	4 (26.7%)	1 (14.3%)	3 (37.5%)	0.57
Resection prior to radiation	15 (93.8%)	7 (100%)	9 (100%)	1.00
Ki-67* (*n* = 9)	13 (7.2–21.3)	13.2 (8.7–13.2)	16.5 (5.5–23.2)	1.00
Skull-base tumors	5 (31.3%)	2 (28.6%)	3 (33.3%)	1.00
Volume radiation field (CM^3^), median, interquartile	26 (6.1–184)	7.5 (2.9–103)	65.4 (16.3–197)	0.14
Isodose prescription	98 (90–100)	99 (75–100)	98 (92–100)	1.00
Radiosurgery	6 (37.5%)	4 (57.1%)	2 (22.2%)	0.302
Radiotherapy	10 (62.5%)	3 (43.9%)	7 (77.8%)	
Median follow-up (months, interquartile)	52 (26–70)	65 (3–74)	44 (34–67)	1.00
Recurrence	8 (50%)	3 (42.9%)	5 (55.6%)	1.00
Time to Recurrence (months, interquartile)	39 (23–56)	40 (32–49)	37 (23–54)	0.77
Radiation Morbidities				
Headache	5 (31.3%)	3 (42.9%)	2 (22.2%)	0.60
Seizure	4 (25.0%)	2 (28.6%)	2 (22.0%)	1.00
Asymptomatic edema	1 (6.3%)	0 (0%)	1 (11.1%)	1.00
Symptomatic edema	5 (31.3%)	2 (28.6%)	3 (33.3%)	1.00
Radiation necrosis	2 (12.5%)	0 (0%)	2 (22.2%)	0.48
Temporal neurological deficit	1 (6.3%)	0 (0%)	1 (11.1%)	1.00
Permanent neurological deficit	0 (0%)	0 (0%)	0 (0%)	
Treatment for Radiation Morbidities				
Steroids	3 (18.8%)	2 (28.6%)	1 (11.1%)	0.55
Antiepileptics	4 (25.0%)	2 (28.6%)	2 (22.2%)	1.00
Surgery	1 (6.3%)	0 (0%)	1 (11.1%)	1.00
Bevacizumab	0 (0%)	0 (0)	0 (0)	

## Discussion

The role of the dural tail in meningioma recurrence is poorly understood, and few studies have sought to determine the clinical relevance of its inclusion in radiation treatment plans. In this study, a total of 187 WHO grade 1 intracranial meningiomas with radiographic evidence of a dural tail were treated with radiation that included or excluded dural tail coverage. Although previous studies suggested that dural tail treatment was associated with a reduction in tumor recurrence rate (15, 16), our study showed that radiotherapy coverage of dural tail did not impact overall rate of recurrence in either SRS or FSRT treated tumors. Further, we did not find that coverage of the dural tail in radiation treatment plans increased the risk of radiation associated side effects.

In our study, we had an overall local control rate of 96.8% for grade 1 meningiomas at a median follow-up of 40 months and 50% in higher grade meningioma at a median follow-up of 52 months. In our SRS and FSRT subgroup analyses, we found similar rates of local tumor control between the dural tail covered and not covered groups. Our results differ from those of Dibiase et al. who assessed recurrence rates based on whether the dural tail was treated or not (15). In univariate analysis of 121 patients, they reported local tumor control (LTC) rates of 96% among those whose dural tail was included versus 77.9% in those without dural tail coverage in their radiosurgery isodose prescription (15). Of note, this study was subsequently refuted by Rogers et al. and others who asserted that the improvement in recurrence-free survival was due to an increase in treatment area, and not necessarily to the inclusion of the dural tail (17). They also raised concern over increased risk for complications of radiation therapy, such as cerebral edema, with the expanded treatment field. Furthermore, Rogers et al. highlighted the critique that Dibase et al. did not provide a clear definition for dural tail. In our study, we used the Goldsher criteria that was independently reviewed by a radiation oncologist and neurosurgeon. In addition, we did not see increased rates of radiotherapy complications with inclusion of the dural tail in the treatment plan. We believe our study adds stronger clarity on the effect of including the dural tail in treatment of meningiomas.

Subsequent studies have brought into question the necessity of including the entire dural tail in stereotactic radiosurgery isodose prescriptions. In 2014, Bulthuis et al. performed an observational study of 203 patients who received SRS to the bulk tumor and only the aspect of the dural tail “closest to the bulk tumor.” The group reported a LTC rate of 96.2%; importantly, no tumor recurrence was determined to have originated from the untreated dural tail (16). This description of tail location was not well described and would be difficult to reproduce clinically. More recently, Lovo et al. reported their experience with treatment of the dural tail in 58 patients with grade 1 meningioma and dural tail sign. At a median follow-up of 3.2 years, they found no statistical difference between tumor control rates of dural tail covered (*N* = 18; local control rate = 95%) versus dural tail not covered (*N* = 38; local control rate = 95.5%) (*P* = 0.574) (18). Although these findings are consistent with those of our study, the small patient size raises concern for an inadequately powered study. Further, this study did not assess for differences in adverse events between the dural tail treated and non-treated groups.

Our study is novel in being the largest formalized investigation of radiotherapy of the dural tail. Our results indicate that there is no benefit to radiating the dural tail, but also that radiation of the tail does not confer increased risk for side effects. Further, our results show that for SRS, radiation of the dural tail may decrease the need for antiepileptics following treatment. This finding may be explained in part by the higher rates of convexity meningioma in the dural tail not covered group (60.0% vs 11.6%, *P* = 0.001), which are more likely to cause symptomatic edema and seizures following radiation (19). This effect would have to outweigh tumor size as the dural tail covered tumors were more than double the size of the non-covered, which should confer greater risk for edema development (20). Certainly, these data provide reassurance for judisciously including the dural tail in radiation fields without increasing the risk of adverse events.

When we assessed the areas of recurrence in 14 recurrent grade 1, 2, and 3 tumors, we found that no tumors recurred from an untreated dural tail, providing further evidence that inclusion of the dural tail does not improve prognosis. Instead, recurrence often occurred from other areas outside of the radiation field suggesting tumor infiltration of other surfaces should be accounted for when making radiation plans. When we considered SRS and FSRT subgroups separately, we once again found that there was no difference in recurrence rate for dural tail covered and not covered in each subgroup, demonstrating that this result is not dependent on radiation method. We obtained similar results when assessing high grade tumors as a cohort, further strengthening our primary finding that treatment of the dural tail does not improve recurrence rate. When considered altogether, our results suggest that radiating the dural tail offers similar recurrence rates but no increased risk for radiation-related adverse effects.

Our study entails a few limitations that need to be taken into consideration. The primary limitation of the study is that although it is the largest of its kind, the sample size is still relatively small. Given that low grade meningioma have a low incidence of recurrence, our sample size and length of follow up may not be sufficient to detect minor differences in recurrence rates and radiation side effects. Furthermore, it is a retrospective study design describing a single-institution experience which introduces the possibility for bias in data collection and means that reproducibility may be limited by our institution's unique clinical practices. Although we attempted to limit bias by blinding the outcome data collectors from those reviewing MRI scans and visa versa, a prospective study will be required to unbiasedly determine the role of radiation of the dural tail. While we utilized three individual reviewers for each patient, the determination of whether a dural tail exists and whether it was included in a radiation treatment plan is variable and subject to interpretation. Another limitation of our study is that we are limited by our follow up of 40 months; it is possible that differences in recurrence rates may have revealed themselves if there was a greater time to follow up. We also had limited statistical power to stratify and assess differences in recurrence rates for grade II and III meningioma given their low number. It is possible that the composition and behavior of the dural tail differs between low grade, anaplastic, malignant and atypical meningioma, such as lipomatous, xanthomatous, or osseous, therefore treatment of the dural tail may need to be catered to the specific tumor phenotype (21, 22). In addition, due to the unavailability of the information for many of our patients, we did not stratify our tumors by Simpson resection grade.

Additional samples will be needed for subgroup analysis and validation of our findings. Future directions in the study of dural tails should include supplementation with new imaging techniques such as 68Ga-DOTATATE PET/CT which have been shown to detect tumor with greater sensitivity than MRI and may help identify tumor containing dural tails (23, 24).

## Conclusion

The results from our study indicate that radiation of the meningioma dural tail does not seem to provide tumor control benefit, but it also does not increase the risk for radiation side effects. A larger radiosurgical registry or prospective randomized study is warranted to fully elucidate the clinical utility and risks associated with radiation of the dural tail.

## Data Availability

The raw data supporting the conclusions of this article will be made available by the authors, without undue reservation.
